# Dissecting genetic pathways in schwannomatosis and malignant rhabdoid tumour

**DOI:** 10.1186/1897-4287-10-S2-A8

**Published:** 2012-04-12

**Authors:** E Algar

**Affiliations:** 1 Molecular Oncology Laboratory, Murdoch Children’s Research Institute, Royal Children’s Hospital, Flemington Rd, Parkville, 3052, Australia

## 

Schwannomatosis is a form of Neurofibromatosis type 2 (NF2) characterized by multiple schwannomas without vestibular involvement, affecting the cranium, spine and periphery. Several recent genetic studies have implicated the *SMARCB1/INI1* tumour suppressor gene in familial schwannomatosis. *SMARCB1* is located centromeric to *NF2* on 22q and loss of function of *SMARCB1* is also a hallmark of malignant rhabdoid tumour (MRT), a highly aggressive tumour of infancy. Both familial and sporadic schwannoma tumours show a mosaic pattern of SMARCB1 protein expression, suggestive of tumour cells either haploinsufficient, or null for SMARCB1 protein. Familial schwannomas linked to constitutional *SMARCB1* mutation can also have somatic mutation of *NF2*, and conversely, schwannoma tumours associated with constitutional *NF2* mutation show mosaic loss of *SMARCB1*, suggesting the involvement of a four-hit mechanism. Molecular analysis for evidence of constitutional SMARCB1 mutation is important in both familial and sporadically occurring schwannomatosis because of the transmission risk for a mutation predisposing to the incurable MRT, in early childhood. However as for NF2, recent evidence suggests that constitutional *SMARCB1* mutations have variable penetrance and exhibit mosaicism, highlighting the importance of examining multiple tumour tissue samples as well as blood in affected individuals to ascertain germline predisposition and to provide accurate counseling for transmission risk. Further studies are needed to define the SMARCB1 mutation spectrum in schwannomatosis and to dissect the strikingly different biology between schwannoma and MRT.

